# Network Visualization and Pyramidal Feature Comparison for Ablative Treatability Classification Using Digitized Cervix Images

**DOI:** 10.3390/jcm10050953

**Published:** 2021-03-01

**Authors:** Peng Guo, Zhiyun Xue, Jose Jeronimo, Julia C. Gage, Kanan T. Desai, Brian Befano, Francisco García, L. Rodney Long, Mark Schiffman, Sameer Antani

**Affiliations:** 1 Communications Engineering Branch, Lister Hill National Center for Biomedical Communications, U.S. National Library of Medicine, National Institutes of Health, Bethesda, MD 20894, USA; zhiyun.xue@nih.gov (Z.X.); rlong@mail.nih.gov (L.R.L.); santani@mail.nih.gov (S.A.); 2 Division of Cancer Epidemiology and Genetics, National Cancer Institute, NIH, Rockville, MD 20850, USA; jose.jeronimo@nih.gov (J.J.); julia.gage@nih.gov (J.C.G.); kanan.desai@nih.gov (K.T.D.); schiffmm@mail.nih.gov (M.S.); 3 Information Management Services, Inc, Rockville, MD 20850, USA; BefanoB@imsweb.com; 4 Health Promotion Sciences Department, University of Arizona, AZ 85724, USA; francisco.garcia@pima.gov; 5 Pima County Arizona, AZ 85701, USA

**Keywords:** cervical cancer, thermal ablation, treatability, deep learning, RetinaNet features, customized CNN, concatenated features, class activation mapping, class relevance mapping, network visualization

## Abstract

Uterine cervical cancer is a leading cause of women’s mortality worldwide. Cervical tissue ablation is an effective surgical excision of high grade lesions that are determined to be precancerous. Our prior work on the Automated Visual Examination (AVE) method demonstrated a highly effective technique to analyze digital images of the cervix for identifying precancer. Next step would be to determine if she is treatable using ablation. However, not all women are eligible for the therapy due to cervical characteristics. We present a machine learning algorithm that uses a deep learning object detection architecture to determine if a cervix is eligible for ablative treatment based on visual characteristics presented in the image. The algorithm builds on the well-known RetinaNet architecture to derive a simpler and novel architecture in which the last convolutional layer is constructed by upsampling and concatenating specific RetinaNet pretrained layers, followed by an output module consisting of a Global Average Pooling (GAP) layer and a fully connected layer. To explain the recommendation of the deep learning algorithm and determine if it is consistent with lesion presentation on the cervical anatomy, we visualize classification results using two techniques: our (i) Class-selective Relevance Map (CRM), which has been reported earlier, and (ii) Class Activation Map (CAM). The class prediction heatmaps are evaluated by a gynecologic oncologist with more than 20 years of experience. Based on our observation and the expert’s opinion, the customized architecture not only outperforms the baseline RetinaNet network in treatability classification, but also provides insights about the features and regions considered significant by the network toward explaining reasons for treatment recommendation. Furthermore, by investigating the heatmaps on Gaussian-blurred images that serve as surrogates for out-of-focus cervical pictures we demonstrate the effect of image quality degradation on cervical treatability classification and underscoring the need for using images with good visual quality.

## 1. Introduction

Uterine cervix cancer is the fourth most common cancer in women with nearly 570,000 new cases reported by the World Health Organization (WHO) in 2018 [1]. The disease is singularly caused by persistent infection with certain oncogenic types of the Human Papillomavirus (HPV) [2]. Mortality due to the disease can be reduced if eligible women are treated prior to the onset of cancer. Treatment eligibility is determined through expert visual assessment of the cervix following a positive screening and histological confirmation of high grade Cervical Intraepithelial Neoplasia (CIN). Screening techniques include a combination of the HPV test [3], cytological assessment, and visual assessment of the cervix treated with weak (3–5%) acetic acid or a solution of Lugol’s iodine. Treatment strategies include cryotherapy, a typical ablative treatment recommended by the World Health Organization (WHO), or thermal ablation. In cryotherapy, a cryoprobe is inserted into a speculum-exposed vagina and placed firmly on the surface of the cervix for 3 min, covering the abnormal tissue (usually twice with a thawing cycle between exposures to freezing gas). Cryogenic gas flows through the instrument, making the metal cold enough within a few minutes to freeze and destroy the tissue. However, its major limitation is the need to transport and store the gas used in the treatment, making it costly and difficult for low and middle resource regions (LMRRs) of the world where the disease is most prevalent. Thermal ablation, also called “thermocoagulation”, is an alternative ablative treatment that is more feasible and easier compared to cryotherapy. Eligible women are treated using a reusable metallic probe that is electrically heated to approximately 100 degrees Celsius for 20–40 s, leading to epithelial and stromal destruction [4]. Multiple exposures may be used as needed. The simplicity of the setup makes it particularly suitable for use in LMRR. However, not all women are eligible for a cervical ablative treatment due to anatomical changes on their cervices or characteristics of the precancerous lesions. Normally, the eligibility for applying thermal ablation is determined by visual examination with/without a colposcopy of cervix after the application of 3–5% acetic acid. The eligibility guidelines for thermal ablation and cryotherapy given by WHO are concluded in Table 1 [4].

Previously, we developed a deep learning method using Faster R-CNN architecture to automatically classify digitized cervix images for cervical precancer screening; this method achieved performance superior to human experts (visual inspection of cervix applied with weak acetic acid, or VIA) on digitized film images collected as part of the of the National Cancer Institute’s Guanacaste Study; we call this automatic approach Automated Visual Evaluation (AVE) [5]. We updated this technique to use RetinaNet [6], an object detection network, on a dataset of cervix images captured using smartphones. We achieved an average AUC (area under curve) above 0.9 over models trained using different training/test data splits [7]. Inspired by the above studies, in this work we investigate the effectiveness of object detection networks, such as RetinaNet, in classifying a digitized cervix image, into: (1) eligible for thermal ablation, denoted as “treatable” (negative), or (2) not eligible for thermal ablation, denoted as “not treatable” (positive). We will denote this classification task as “treatability classification” in the rest of this paper. We develop the treatability classification approach, to provide support about treating or patient referring to those providers when the eligibility for ablation could be in question. The approach provides guidance to the providers who might have difficulties accessing well-trained VIA/ ablation health care workers. The approach aims at reducing the (1) false negative cases where CIN3 can potentially transform into cancer with the next year(s); and, (2) controlling false positive cases where treatment could be considered unnecessary. However, in false positive cases, recall that the women referred for evaluation are HPV+. Ablative treatment in HPV+ women decreases the rate of persistent infections, thereby decreasing the risk of those women to be chronically infected and risk of future cancer.

**Figure 1:**
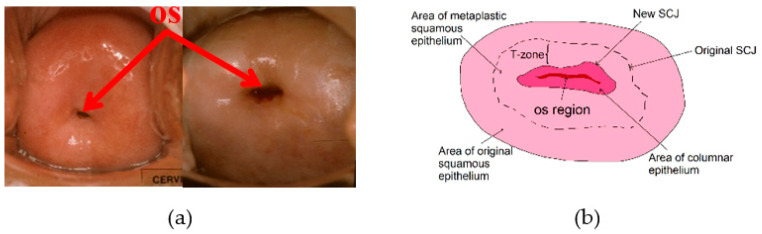
Examples of digitized cervix images and anatomical illustration of a cervix. (**a**) An example of os (the opening into the uterus) having different shapes. (**b**) An illustration of cervix anatomy and illustration of T-zone. The cervix squamous and columnar cell regions are separated by the squamocolumnar junction (SCJ). As a woman ages the SCJ migrates from its original location toward the os.

There are two key issues that need to be researched and investigated in greater depth. Firstly, in the previous AVE and its related studies [7], we noted variable classification performance across sets of cervix images acquired from different geographical locations, which have potentially varying imaging devices (different models/generations of common off-the-shelf smartphones, specialized devices such as MobileODT’s EVA system, or digital Single Lens Reflex (SLR) cameras), environmental factors (e.g., lighting, provider training), and imaging procedures (e.g., handheld smartphones, adapter mounted or colposcope-attached SLR cameras, and time of exposure after application of weak acetic acid). Further, these image datasets differ with respect to image quality factors such as partial/full absence of the anatomical region of interest (cervix), or other factors such as illumination or focus, which could impact classification prediction performance. The effects of these variability and quality factors on the algorithm performance are critical translating classification into clinical guidance or subsequent use in treatability classification. Secondly, since deep learning classifiers are considered “black box” decision-makers, it is imperative to explain classifier decision-making for meaningful clinical guidance. This is done using visualization of internal convolutional layers superimposed on the cervix to evaluate consistency with the anatomy and disease presentation. The os (also called “external os”, shown in Figure 1a,b) and the transformation zone (T-zone or TZ) (Figure 1b) are regions that are of clinical importance for diagnosing precancer and making treatment decisions [8], because most cervical abnormalities are found to develop in these regions [9]. It is important to understand whether the model bases its classification score based on their appearance. This could potentially be answered by understanding the relationship between the deep learning features and the classification output through visualization techniques.

Deep network visualization and interpretation is a research area of growing interest [10,11,12,13,14,15,16,17,18,19]. A survey of various techniques is given in [20]. Representative methods based on Convolutional Neural Networks (CNNs) include: saliency mapping, which calculates the gradient of the output score with respect to each pixel in the input image; Local Interpretable Model-Agnostic Explanations (LIMEs) [13], which evaluates the importance of image components (superpixels obtained from a standard algorithm) with respect to the linear regression coefficients obtained using perturbed image samples as input; and Class Activation Maps (CAMs) [14], which computes the linear combination of the activations of the last convolutional layer in CNNs with respect to class output and requires the model to have specific layers such as a Global Average Pooling Layer (GAP) and a Fully Connected (FC) layer at the output. We have previously reported our own visualization method called the Class-selective Relevance Map (CRM) [21]. In CRM, a feature map element (one element in the feature matrix) in the last convolution layer is considered to be significant if removing that element leads to a significant increase in the overall Mean Squared Error (MSE) in the classification prediction. This overall error is the sum of MSE between the prediction and the target for each node in the output layer. Therefore, CRM takes both positive and negative contributions (i.e., contribution toward an increase in the output score for the desired class and a decrease in the scores for the other classes) for each element in the feature maps into consideration.

Note that these visualization methods have been developed mainly for the interpretation of deep learning networks, which assign a class label to an entire input image. These methods cannot be directly applied to deep object detection networks, such as RetinaNet, since the outputs of such networks include not only the classification confidence score for each detected object, e.g., the cervix or the os, but also a confidence score for its location on the image. In addition, object detection networks merge initial predictions of object regions to generate final regions using Non-Maximum Suppression (NMS), which makes the direct visualization of the object detection network very difficult.

To address this issue, we incorporate the object detection network into a classification network by using the pyramidal feature layer(s) from RetinaNet and appending a GAP layer and a FC layer with softmax activation at the output. Next, we train this new classification model by keeping the features obtained from the originally trained RetinaNet model unchanged. By visualizing attention regions of this new model using CAM and CRM, we are able to gain insight into our network’s classification decisions, and also the decisions of the original RetinaNet model.

The Feature Pyramid Network [6] used in the RetinaNet model computes features of different scales from the ResNet50 [22] backbone these differently scaled features potentially contribute in the object detection tasks where multiple target objects of varying sizes exist. However in our case, there is only one major target object, the cervix. To investigate which pyramidal feature or their combination deliver the best classification and localization performance, we decompose the Feature Pyramid Network (FPN) and build corresponding classification networks and visualize their mapping between feature layer and classification outputs. The details of the classification networks’ architectures are described in Section 3.1.

To evaluate the performance of the two visualization techniques on our cervix images, we generate the heat maps by applying CRM and CAM methods on our best-performing classification model. Next, the heatmaps are manually evaluated by a gynecologic oncologist expert and the comments on heatmap placement and comparison of the two visualization methods (namely, CRM and CAM) are collected. We present our analysis based on our models’ performance and the expert’s feedback. We find that:

- The model built with P3, P6, and P7 concatenated as the last convolutional layer achieves better classification performance than other models.

Additionally, referring to the human expert’s evaluation:

- The heatmaps from both CRM and CAM shows that the most relevant image pixels for making correct classification are those in or around the os and T-Zone.
- Compared to CAM, the CRM (1) visualizes and focuses more on the area around the os and T-zone; and (2) generates fewer heatmaps that the human expert disagrees with.

In addition to the evaluation of the heatmap placement, the expert points out that:

- In the review, the expert has opposite opinions about ground truth of several images, and observes incompleteness of cervix display in some images samples. These issues need further attention since they can lead to misclassifications leading to incorrect treatment decisions.

Lastly, we apply three levels of Gaussian blur on our test images and test our best-performing model on those blurred images. The observed classification performance drop and the misplacement of the corresponding heatmaps indicate that:

- Good image/object quality is a key factor to ensure correct classification, and quality degradation can be a huge distractor for capturing significant features and for making correct classifications.

The rest of the paper is organized as follows: Section 2 describes the details of datasets used in this study, Section 3 describes the network architecture and two visualization methods; Section 4 and Section 5 present the experiments, the results and the conclusion.

## 2. Image Data

**Figure 2:**
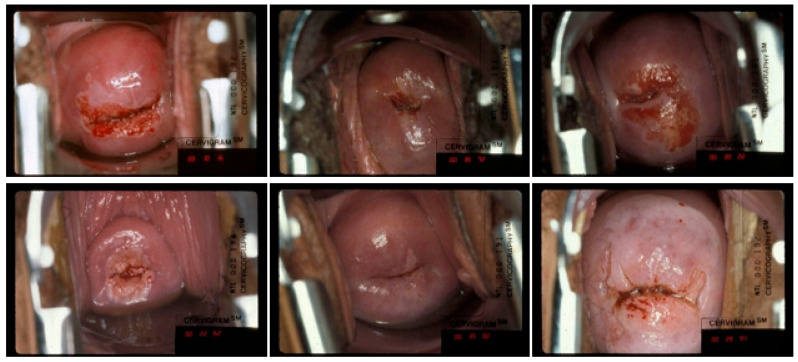
Examples of cervix images used in this study, the top row images are labeled as “treatable” and the bottom row images are labeled as “not treatable” (reasons for not treatable given by the expert: left—lesion in canal, middle—SCJ not visible, right—lesion too large for ablation).

The images used in this study were selected from the longitudinal cohort study (conducted from 1993 to 2001) of HPV and cervical cancer, provided by the NCI-funded Proyecto Epidemiologico Guanacaste. During the study, approximately 30,000 screening visits were recorded each of which included multiple kinds of tests (cytology, HPV testing, and cervicography). Cervicography is a visual screening method based on the interpretation of a pair of cervical photographs (called cervigrams) [23,24]. The cervigrams were taken at each screening visit using specially equipped camera called a Cerviscope. The obtained photographic images were later presented via a software tool developed by the National Library of Medicine and the National Cancer Institute to two highly experienced human experts who labeled the cervix in the images as “treatable” or “not treatable” [25]. The cases in which the experts disagreed are discussed and resolved by consensus. After the completion of the labeling efforts, 1033 images, which the expert considered as clear cases of “treatable” (729 images) or “not treatable” (304 images), are selected to form the dataset used in this study (samples shown in Figure 2). We randomly selected approximately 80% of the “treatable” and “not treatable” images as the training images, and the remaining approximately 20% images as testing images. We carried out our random selection on the “woman level”; that is, we did not allow the image(s) belonging to the same woman to be in both the training and testing sets.

## 3. Methods

### 3.1. Network Architecture

**Figure 3:**
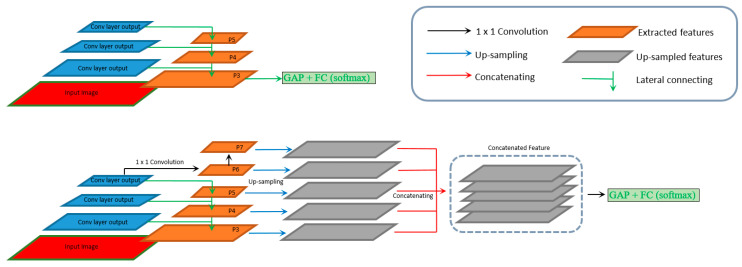
High-level architecture of the feature pyramid and our fine-tuned classification models with global average pooling (GAP) and fully connected (FC) layers. Top figure shows the architecture using one single pyramidal feature as the last convolutional layer, bottom figure shows the customized architecture using concatenated pyramidal features as the last convolutional layer. As each RetinaNet feature has 256 channel, the concatenated feature has 256 × N (feature number) channels. As shown in the 5 stacked gray feature blocks above, the concatenated features (P_3_, P_4_, P_5_, P_6_, and P_7_) have 1280 channels.

RetinaNet [6] computes features using a FPN [26], where features are extracted at different blocks of ResNet50 deep learning backbone architecture and then resampled and merged via lateral connections (top-down pathways as shown in Figure 3). Since RetinaNet is a one-stage object detection network, classification decisions are made based on the probability of each spatial location belonging to a particular category. In order to convert it to a classification network, which outputs a classification score for the entire input image, we replace the classification and regression subnetworks in RetinaNet at all locations with a single 256-channel GAP layer and a FC layer with a two-class softmax output. From the FPN, we select 5 pyramidal layers: (1) P5, the 1 × 1 convolutional output of C5 in ResNet50; (2) P6, the 3 × 3 convolutional output of C5 in ResNet50; (3) P7, the 3 × 3 convolutional output of P6; (4) P4, the 3 × 3 convolutional output of M4 (the weighted sum of up-sampled P5 and 1 × 1 convolutional output of C4 in ResNet50) and (5) P3, the 3 × 3 convolutional output of M3 (the weighted sum of up-sampled M4 and 1 × 1 convolutional output of C4 in ResNet50). In total, we are able to create seven models by building their last convolutional layers in two ways: (a) select only one of the five layers as the last convolutional layer (top figure in Figure 3), or (b) upsample the low dimension feature layer(s) and concatenate that with other high dimensional feature(s) (bottom figure in Figure 3). Either way, the generated convolutional layer is connected to a GAP and a FC layer. Each model is separately trained with shared pretrained features from the RetinaNet model. This allows us to apply the visualization methods such as CAM and CRM, without changing the features in the object detection network, which include both spatial and intensity information contributing to the identification of a particular category.

### 3.2. Class Activation Map (CAM)

CAM [12] is a popular visualization method for CNNs. It requires a specific model architecture in which convolutional features are global average pooled and then fed into a fully connected layer. The output score $s_{c}$ for a category “c” (treatable or not treatable) can be expressed as the weighted sum of the global average pooling:

(1) $$s_{c}=\sum_{K}w_{k}^{c}F^{k}=\sum_{K}w_{k}^{c}\sum_{x,y}f_{k}\left(x,y\right)=\sum_{x,y}\sum_{k}w_{k}^{c}f_{k}\left(x,y\right),$$

where $w_{k}^{c}$ denotes the weight for the k-th feature map corresponding to class “c”, $F^{k}$ represents the global average pooling and $f_{k}\left(x,y\right)$ is the spatial activation at location (x, y), which is called L henceforth. Thus, the CAM of a specific class c can be formulated as the sum of the spatial activation across all the feature maps:

(2) $$M_{c}\left(x,y\right)=\;\sum_{k}w_{k}^{c}f_{k}\left(x,y\right),$$

In other words, CAM reflects the importance of the activation at each spatial element at L for the classification of an input image to class c. By simply upsampling the CAM to the size of the input image, we located the Region-of-Interest (RoI) within the image that contributes the most to the particular category.

### 3.3. Class-Selective Relevance Map (CRM)

Compared with CAM, our Class-Selective Relevance Map (CRM) includes the concept of “relevance” [27]. As presented in [21], the relevance of a hidden node is computed as the incremental MSE at the output layer, but without that node. A hidden node with a large relevance score could be important to the classification decision, since removing that specific node will lead to a significant increase in MSE. The CRM uses this idea by removing the value at L in the feature maps from the last convolutional layer, and calculating the before-and-after (removal) MSEs using Equation (1). The CRM score at L is calculated from all nodes in the output layer as:

(3) $$M_{c}\left(x,y\right)=\sum_{c=1}^{N}{\left\{\left(s_{c}-\;s_{c}\left(x,y\right)\right)\right\}}^{2},$$

where $s_{c}$ denotes the classification score and $s_{c}\left(x,\;y\right)$ denotes the score after setting the value at that location to zero. An important feature element should: (1) make positive contribution to the increase of the prediction score at the output node representing the desired class and (2) make negative contribution to the decrease of the prediction score at the other output nodes. In contrast, CAM only considers the prediction score for the particular class to which a given input image is assigned.

## 4. Experiments

### 4.1. Classification Performance

As discussed in Section 3.1, we initially did fine tuning to obtain classification models having the last convolutional layer built from one or several of the pyramidal feature layers: $P_{3}$, $P_{4}$, $P_{5}$, $P_{6}$, and $P_{7}$ [6]. All the models used the same hyper parameters for training except the final selected epoch number for the best model. All images were resized to have the longer border to be 1200 pixels, maintaining aspect ratio. The base network was ResNet50 [22]. The models were trained with a learning rate of 1 × 10^−6^ using a GeForce 2080 Ti GPU. The weights of the convolutional feature layers were from the RetinaNet model and remained frozen in the training; the weights of the added fully connected layer were randomly initialized. The pixel values in the input image were rescaled to be within (0, 1).

We evaluated the classification performance in terms of accuracy and F1-score as shown in Table 2. The highest accuracy of 86.47% was obtained for classifying the cervix images into “treatable” and “not treatable”, from the classification model trained with $P_{3}$, $P_{6}$, and $P_{7}$ as the last convolutional layer. The model using concatenated features ($P_{3}$, $P_{6},\;\mathrm{and}$ $P_{7}$) outperformed our pre-trained RetinaNet model (accuracy = 0.8011), and it had a much simpler architecture.

### 4.2. Pyramidal Feature Comparison

As shown in Table 2, the classification model with $P_{3}$, $P_{6}$, and $P_{7}$ as the last convolutional layer achieved the best classification result among the seven models shown in Table 2. The concatenated features ($P_{3}$, $P_{4}$, $P_{5}$, $P_{6}$, and $P_{7}$) achieved the second best, while the results obtained with single layer $P_{4}$ and $P_{5}$ as the last convolutional layer were less satisfactory. To further examine their visualization, we applied CRM and CAM to all the seven models with test images as input. We would like to demonstrate the heatmaps in two ways with respect to the pyramidal feature component(s) of the last convolutional layer in the classification models: (1) single pyramidal feature used as the last convolutional layers, such as $P_{3}$, $P_{4}$, $P_{5}$, $P_{6},$ or $P_{7}$. These features are extracted from the RetinaNet model, which achieved significant classification performance. By visualizing these models, we are able to gain insights into our fine-tuned models’ classification decisions, and also the decisions of the classification subnet in the RetinaNet model; and (2) multiple pyramidal features concatenated as the last convolutional layer, such as the combination of ($P_{3}$, $P_{6}$, $P_{7}$) and ($P_{3}$, $P_{6}$, $P_{7}$, $P_{3}$, $P_{6}$, $P_{7}$). To construct such convolutional layers, we upsample and concatenate multiple feature layers in the pretrained RetinaNet so that the classification is made taking all the concatenated features into consideration. This is different from the scenario in the original RetinaNet where the classification subnet classifies each pyramidal feature separately. Since the concatenated features deliver higher performance as shown in Table 2, we are also interested in looking into the heatmaps generated based on these features. Note that the heatmaps are generated after applying the threshold at 20% of the maximum classification score and normalizing the associated pixel values of detected image regions into the range (0, 255). In the heatmaps, warmer colors (i.e., more reddish and yellowish colors) correspond to greater significance of the region in the classification decision.

#### 4.2.1. Single vs. Concatenated Pyramidal Features

**Figure 4:**
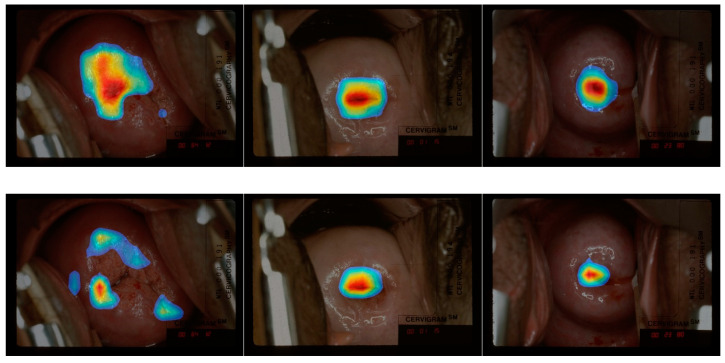

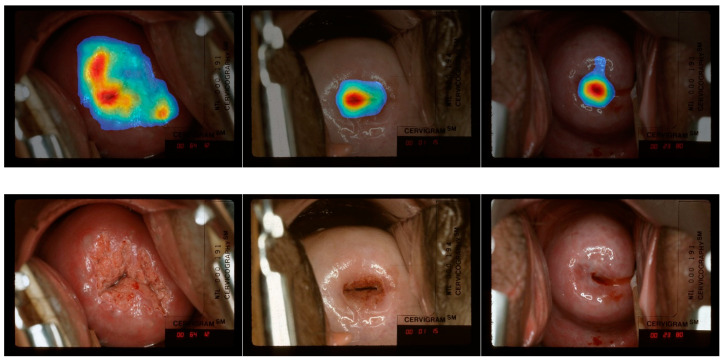
Example of Class-selective Relevance Map (CRM) heatmap images generated from the fine-tuned classification model. The heatmaps in the 1st row are generated from the classification model built with Concatenate (*P_3_*, *P_6_*, *P_7_*) as the last convolutional layer. The heatmaps in the 2nd row are generated from the classification model built with P_6_ as the last convolutional layer. The heatmaps in the 3rd row are generated from the classification model built with Concatenate (*P_3_*, *P_4_*, *P_5_*, *P_6_*, *P_7_*) as the last convolutional layer. The bottom row images are without any heatmap displayed.

In Figure 4, the first row shows the examples of heatmap images obtained from the model with concatenated features ($P_{3}$, $P_{6}$, $P_{7}$) as the last convolutional layer, given input images are listed correspondingly down at bottom. Our attention was paid on the heatmap placement near the os and TZ, which, based on the WHO guideline (Table 1), were highlighted with significance for classifying a cervix to be ablative “treatable” or “not treatable”. It can be observed that the CRM consistently highlighted the region inside the cervix, our primary region of interest. More specifically, the CRM highlighted the region on top of or around the os, which appeared as a concave dark area in the image. In some examples (images in the first row in Figure 4), the CRM visualization highlights the area that is part of the “T-zone”. These observations support our expectation that clinically important regions, such as the “T-Zone”, may contribute the most to the treatability classification. The heatmaps obtained from the model with single pyramidal feature as the last convolutional layer, $P_{6}$ for example (second row images in Figure 4), show similar attention around the os. However, the visualization sometimes highlights areas that is insufficient and off the expected area (1st image in the second row in Figure 4).

#### 4.2.2. Concatenated vs. Concatenated Pyramidal Features

Although the concatenated features ($P_{3}$, $P_{4}$, $P_{5}$, $P_{6}$, $P_{7}$) consist of five pyramidal features, the model trained using them underperforms the model using only three pyramidal features ($P_{3}$, $P_{6}$, $P_{7}$). As shown in our visualization examples in Figure 4, the CRM highlights similar area when using two models (1st and 3rd images in row 1 and 3 of Figure 4). However, the heatmaps of the model using ($P_{3}$, $P_{4}$, $P_{5}$, $P_{6}$, $P_{7}$) sometimes exhibited much larger highlighted region (as shown in 1st image in row 3 of Figure 4).

### 4.3. Expert Evaluation of Heatmaps

To evaluate the performance of the visualization techniques on our cervix images, we generated the CRM and CAM heatmaps based on the outputs of our best performing model ($P_{3}$, $P_{6},$ and $P_{7}$ concatenated). We asked a medical expert (a gynecologic oncologist with many years’ experience in specializing in cervical screening and outpatient treatment) to review the correctly classified images with corresponding heatmaps generated by both visualization techniques. The expert’s opinions and comments regarding the visual observation of the CRM/CAM heatmap placement were collected. In addition, for each reviewed image labeled as “not treatable”, one/several specific reason(s) was/were given by the expert. All the given reasons can be found as the referral criteria listed in Table 3. These referral criteria include more specific factors and descriptions compared with the WHO guideline and are on the efforts of being proposed as a standard for judging a cervix as “not treatable” and referring to colposcopy. The information provided by the expert is very important in assisting us to understand the network output and correlate the heatmap representation with clinical explanations. Using these criteria, we are able to examine whether the decision/heatmap is reasonably made/placed with respect to the given reason(s) and the expert’s opinions. In the expert’s review of all the 897/1033 correct classified images and the corresponding heatmaps, 74/83 (CRM/CAM) heatmaps are labeled as “misplacement”. These heatmaps are considered: (1) having highlighted area out of the area of interest, (2) having insufficient coverage of RoI, (3) having incorrect ground truth label, or (4) being bad images or images with insufficient quality.

#### 4.3.1. CAM vs. CRM Heatmaps

**Figure 5:**
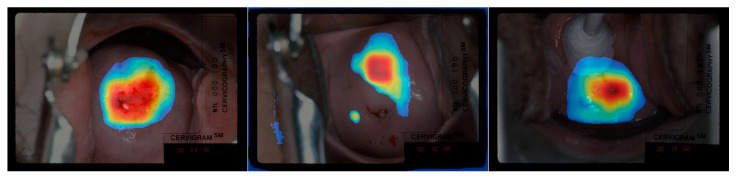

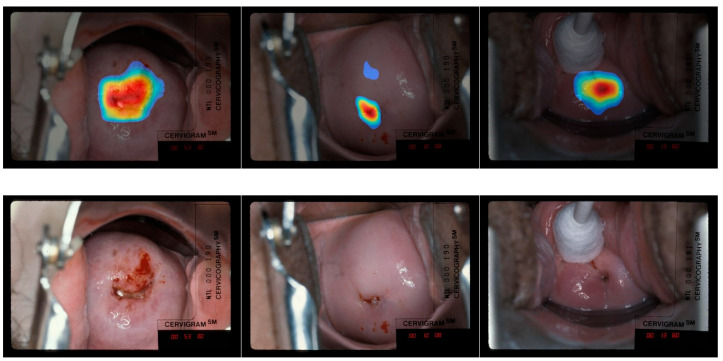
Top: CAM visualization of model built with concatenated features (*P_3_*, *P_6_*, *P_7_*) as the last convolutional layer. Bottom: CRM visualization of the same model as top. Note that the 1st and 2nd column from the left are “treatable” images, 3rd is the “not treatable” images (reason given by human expert: the SCJ is not visible, so the network is expected to look at the os region). Original images without heatmaps are shown in the 3rd row, respectively.

As visualized in Figure 5, the differences between CRM and CAM heatmaps were slightly larger among images correctly classified as “not treatable” as compared to images correctly classified as “treatable”. Based on the expert’s opinion from comparing CAM and CRM heatmaps, our judgment is that CRM heatmaps generally contain less irrelevant area and highlight significant areas with more constrained boundaries. We obtained a quantitative comparison by calculating the ratio of the number of highlighted pixels over the entire feature map in CAM and in CRM, and found that the RoI size in CRM is 20% less than that in CAM on average. This could be explained by the different mechanisms used to generate the heatmap values in these two methods: CAM uses the weighted sum of a feature map, while CRM calculates MSE and rejects elements in the feature map if they have negligible influence in making the correct classification. There are also some examples showing that the CRM heatmap is labeled as “meaningful” while the corresponding CAM heatmap is labeled as “out of the region of interest” by the medical expert (2nd column in Figure 5). Overall, over 90% of the heatmaps generated by CRM and CAM visualization techniques are considered “meaningful” by the medical expert. We consider that (1) the CRM and CAM are having similar performance based on the expert’s comments and (2) CRM performs slightly better than CAM since there are less number of CRM heatmaps that the expert disagrees with.

#### 4.3.2. Out of Region of Interest (RoI) and Insufficient Coverage

**Figure 6:**
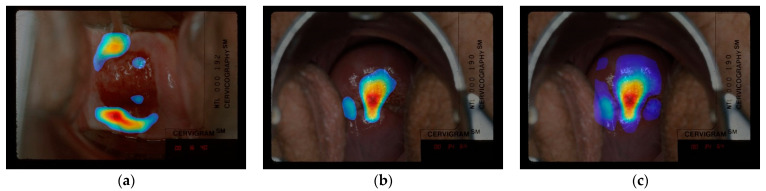
(**a**) CRM visualization, which is considered as “highlighted area out of the region of interest”. (**b**) CRM visualization, which is considered as “insufficient coverage of region of interest”. (**c**) Sample of CRM visualization with a lower threshold of 15% of the maximum score on the same image with (**b**).

For the heatmap placement that the expert disagrees with, “highlighted area is out of the region of interest” (left image in Figure 6) and “the heatmap has insufficient coverage of the RoI” (middle image in Figure 6) are the two comments that are majorly associated. The former, is describing the heatmaps in which the landmark pixels (such as the os) are not highlighted. As we observed, these images are labeled with reasons (such as “cervix distortion”, “vaginal wall close”, “lesion too large” or “suspect invasive or glandular disease”), which are associated with a few (around 5%) image samples. The misplacement of heatmaps can be explained by lack of training sample with these specific factors. The later comments, “insufficient coverage of the region of interest”, is given when the highlighted area borders are constrained within, but not close to the Squamous Columnar Junction (SCJ), which the expert uses to locate a visible TZ. The heatmap is expected to exhibit a full coverage within the SCJ when a TZ is visible. This could be alleviated by tweaking the threshold value, which is set by default at 20% of the maximum score, the before-and-after comparison is shown in Figure 6 (before: middle image, after: right image).

#### 4.3.3. Inaccurate Ground Truth Label and Bad Image

**Figure 7:**
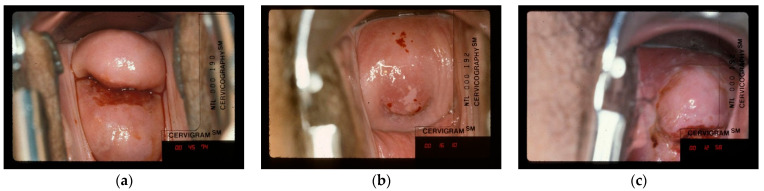
(**a**) Image sample labeled as “treatable” but considered as “not treatable” by the human expert. (**b**,**c**) Image samples that the human expert considers as “bad image” (the full cervix is not visible and bad focus).

There are images that the expert considers as “labeled with incorrect ground truth” (Figure 7a), which indicates the expert has opposite opinion against the ground truth label, which is previously labeled in a separate evaluation. Additionally, we observed that the expert gives comments of “bad image” on some samples (two examples shown in Figure 7b,c), which either are blurry or fail to show a complete view of the cervix. As the major RoI in this task, the complete and clear display of the cervix is necessary to the identification of “treatable” or “not treatable”. These images with either controversial ground truth or with insufficient quality, from a machine learning perspective, can potentially harm the model’s classification performance and visualization results. The “inaccurate ground truth” is non-remedial at the image processing level, since it is caused by the data acquisition operations that are completed before we have the images. However, for the images with insufficient quality caused by blurriness or out-of-focus, we can try corrective measures; for example, we have developed deep learning techniques [28] to filter out such bad images and to recover them to a certain degree.

### 4.4. Quality Degradation

**Figure 8:**
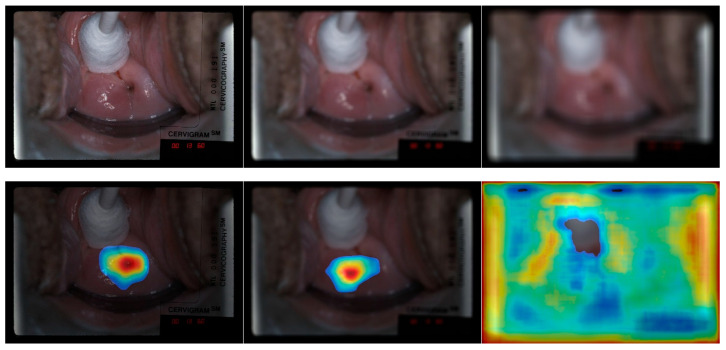
Example of CRM heatmap generated from the fine-tuned classification model built with concatenated features (*P_3_*, *P_6_*, *P_7_*) on blurred images. The top row images are input images: left—original unblurred, middle—blurred with (19, 19) filter size, right—blurred with (49, 49) filter size. The bottom row images are heatmaps generated using the left, middle and right images in the top row, respectively. In the heatmap of the original unblurred image, the highlighted area is around the os region, the middle heatmap although visually looks similar with the left one, the highlighted area is off the os region.

We also investigated the effect of quality-degraded images on classification performance and visualization results. We generated degraded images by applying Gaussian blur using filters of size (19, 19) and (49, 49) (as shown in Figure 8 row a). This degradation serves as a surrogate for out of focus pictures that are often the bane of automated cervical screening. We aim to observe (1) classification robustness with respect to the level of degradation, and (2) the changes in CRM visualization results. As shown in Table 4, and as expected, quality degradation does lead to a drop in classification performance. Although the filter sizes for the Gaussian blur are much smaller than the image size, the negative effect on the classification performance is significant: as much as 50.15% with the largest filter size used. This negative effect could be potentially much more severe in a real scenario. With respect to visualization, as shown in Figure 8, the highlighted regions are either aggressively “growing” into the surrounding area/even the entire image (right image in the bottom row of Figure 8) or relocated to other questionable positions (middle image in the bottom row of Figure 8) when increasing the level of degradation. These findings underscore for acquiring and presenting good quality images to the classifier, and perhaps an automated tool to guide image capture and assess image quality.

## 5. Conclusions

We developed a novel one-stage object detection network for classifying a digitized cervix image as thermal ablation “treatable” or “not treatable” based on the RetinaNet architecture, which achieved very good performance. We investigated the problem of providing some interpretation and explanation of how the model is making classification decisions. Due to the challenges of interpreting the object detection network directly, we propose an indirect approach, which can take advantage of existing visualization methods. Specifically, we build a classification network by migrating the features from the RetinaNet model and by appending global average pooling and fully connected layers. Proceeding in this fashion, we trained, tested, and compared seven models with different pyramidal feature as the last convolutional layer. We used two network visualization methods; one of which is the commonly used CAM, while the other was our method, called CRM.

In this proof-of-principle work, we conducted multiple experiments and provide quantitative classification results, and qualitative evaluations of visualization results based on our judgment about cervix locations of clinically significant regions. An expert gynecologic oncologist with many decades experience reviewed the heatmaps generated by both CAM and CRM methods. Analysis of this review revealed several important findings: (1) the concatenated FPN features of *P_3_*, *P_6_*, and *P_7_* is the optimal feature solution for our classification model; this generates the best classification performance and the most meaningful visualization; (2) both CAM and CRM visualizations are able to reveal useful information, and in most cases they consistently highlight the regions of the os and T-zone, two clinically important regions within the cervix, for making correct AVE classifications. Compared with CAM, CRM appears more capable of excluding irrelevant regions; (3) by comparing the heatmaps obtained with respect to two classes (thermal ablation “treatable” and “not treatable”), we confirm that the models make reasonable classification decisions; (4) images with controversial ground truth labels can be problematic and needs further attention; and (5) insufficient image quality such as blurriness and out-of-focus lead to degradation of the model performance, as demonstrated through the analysis of applying image quality degradation on the tested images. Visual interpretations obtained by comparing CRM visualization between the blurred images and the original images also verify that degradation of image quality can be a significant factor for the loss of important features used in making correct classification.

These findings provide information that is useful in optimizing the classification model, visualization techniques for explanation and interpretation, and data acquisition. More importantly, they highlight the difficult problem of treatability classification and how our AVE [5] screening model could be used in conjunction for appropriate treatment guidance.

## Figures and Tables

**Table 1:** WHO guideline: eligibility for thermal ablation and cryotherapy.

Eligibility	Description	Guideline
treatable	i. screen positive ii. without suspicion of invasive or glandular disease (i.e., adenocarcinoma or adenocarcinoma in situ)	the transformation zone (TZ) is fully visible, the whole lesion is visible and it does not extend into the endocervix, or the lesion is type 1 TZ; or the lesion is type 2 TZ where the probe tip will achieve complete ablation of the SCJ epithelium, i.e., where it can reach the upper limit of the TZ. Sometimes the SCJ can be seen high in the canal but a probe tip would not reach it.
not treatable	i. screen positive ii. with suspicion of invasive or glandular disease (i.e., adenocarcinoma or adenocarcinoma in situ)	the TZ is not fully visible because it is endocervical (Type 3 TZ); or it is a Type 2 TZ where the SCJ is out of reach of the probe tip.

**Table 2:** Classification results for the networks using $P_{3}$–$P_{7}$ as the last convolutional layer. Each of the seven models shown is trained separately for the treatability classification task.

Last Convolutional Layer	Accuracy	F1-Score	Last Convolutional Layer	Accuracy	F1-Score
$P_{3}$	0.7440	0.6241	$P_{7}$	0.7573	0.6212
$P_{4}$	0.6748	0.5315	$P_{3}$, $P_{6}$, $P_{7}$	0.8647	0.7586
$P_{5}$	0.7184	0.5672	$P_{3}$, $P_{4}$, $P_{5}$, $P_{6}$, $P_{7}$	0.8495	0.7156
$P_{6}$	0.8116	0.7111			

**Table 3:** Proposed referral criteria for judging a cervix as “not treatable” [25].

Referral Criteria
**Reasons related to lesions**
Lesion extend into endocervical canal
2. Lesion extends into the fornix
3. Lesion too large for ablation
4. Suspect invasive or glandular disease
**Reasons not related to lesion characteristics**
5. Type 1 TZ—Ectopy too large for ablation
6. Type 2 TZ—TZ is partially endocervical but fully visible (and SCJ is out of reach of probe tip)
7. Type 3 TZ—TZ extends out of view up the endocervical canal.
8. Cervix distorted
9. Vaginal wall close
10. Large polyp

**Table 4:** Classification accuracy for different level Gaussian blurred images.

Last Convolutional Layer	Filter Size/Classification Accuracy	
*P_3_*, *P_6_*, *P_7_*	(19, 19)/67.15%	(49, 49)/36.32%

## Data Availability

Image data used in this study may be available by special request, addressed to Dr. Mark Schiffman (NCI).
